# Assessment of the simultaneous effect of hypothyroidism and thyroid autoimmunity with gestational diabetes on the incidence of type 2 diabetes

**DOI:** 10.1186/s12902-020-00627-z

**Published:** 2020-10-01

**Authors:** Maryam Zahedi, Elham Kazemian, Fahimeh Ramezani-Tehrani, Maryam Tohidi, Fereidoun Azizi, Davood Khalili, Maryam Rahmati, Atieh Amouzegar

**Affiliations:** 1 Endocrine Research Center, Research Institute for Endocrine Sciences, Shahid Beheshti University of Medical Sciences, P.O. Box: 19395-4763, Tehran, Iran; 2 Reproductive Endocrinology Research Center, Research Institute for Endocrine Sciences, Shahid Beheshti University of Medical Sciences, P.O.Box: 19395-4763, Tehran, Iran; 3 Prevention of Metabolic Disorders Research Center, Research Institute for Endocrine Sciences, Shahid Beheshti University of Medical Sciences, Tehran, Iran; 4 Department of Epidemiology and Biostatistics, Research Institute for Endocrine Sciences, Shahid Beheshti University of Medical Sciences, Tehran, Iran

**Keywords:** Gestational diabetes, Hypothyroidism, Tehran thyroid study, Thyroid autoimmunity, Type 2 diabetes

## Abstract

**Introduction:**

Despite the evidence available on the adverse impact of gestational diabetes (GDM) and thyroid disorders on developing type 2 diabetes (T2DM), the concurrent influence of these disorders on the incidence of T2DM has not been reported yet.

**Methods:**

In this prospective study, 1894 non-diabetic women aged 20 to 60 years, with a history of at least one term delivery, without diagnosed hyperthyroidism were selected at the initiation of the Tehran Thyroid Study (TTS). Pooled logistic regression analyses were used to investigate the association of GDM, thyroid disorders i.e., hypothyroidism and/or thyroid peroxidase antibody (TPOAb) positivity and interaction between GDM and thyroid disorders with the risk of incident T2DM.

**Results:**

Of the 1894 participants of the present study, 346 (18.3%) had a history of GDM, and 832 (43.9%) had thyroid disorders. The total cumulative incidence rate of T2DM at the median follow-up time of ~ 12 years was overall 12/1000 person-years (95% confidence interval (CI): 10/1000–13/1000), with an incidence rate of 16/1000 (95%CI: 13/1000–20/1000) in women with GDM; and 11/100,000 (95%CI: 9/100,000–12/1000) among those without GDM. After adjustment for age, the risk of incident T2DM increased among individuals with the previous GDM compared to women without a history of GDM (odds ratio (OR): 1.54, 95%CI: 1.06, 2.25). No significant associations were found between either thyroid disorders or the interaction between GDM and thyroid disorders with the development of T2DM; (OR: 1.14, 95%CI: 0.82, 1.58) and (OR: 1.27, 95%CI: 0.66, 2.43), respectively.

**Conclusion:**

GDM and thyroid disorders have no concurrent impacts on the incidence of T2DM.

## Background

About 10% of pregnancies develop gestational diabetes mellitus (GDM) [1], which causes multiple adverse fetal and maternal health outcomes [2]. The risk of developing type 2 diabetes mellitus (T2DM) among women with a history of GDM is 2–10 fold greater compared to those with a healthy pregnancy [3]. Similarly, thyroid disorders, the most common endocrine disorders in women of childbearing age [2], are more prevalent in pregnant women with GDM compared to their healthy counterparts [4]. Also, a significant increase in the incidence of thyroid autoimmunity has been reported among women with a history of GDM (31.6% vs. 9.7%) [1]. It has also been shown that hypothyroidism is more common among women with T2DM, compared to non-diabetic women [5, 6], e.g., in a population-based prospective cohort study with 7.9-year follow up of 8452 individuals, hypothyroidism was reported as a risk factor for the incidence of T2DM, particularly among women with prediabetes [6]. Although the presence of T2DM and obesity was associated with the increased thyrotropin (TSH) concentration, the causal link between obesity and free triiodothyronine [FT3] and free thyroxin [FT4] is still under debate [7, 8]. Therefore, it should be noted that the association of obesity and T2DM with hypothyroidism remains a gray area because subclinical hypothyroidism might well be secondary to obesity and T2DM [9].

There is a growing body of evidence to support the strong positive association between the presence of GDM and the subsequent risk of developing T2DM [10, 11]. However, the most important and controversial viewpoint is that if thyroid disorders, i.e., hypothyroidism and/or thyroid autoimmunity, have an intermediatory effect in the link between GDM and incidence of T2DM. Nevertheless, previous studies did not consider the concurrent impact of the past history of GDM and thyroid disorders, i.e., hypothyroidism and thyroid autoimmunity, on the risk of developing T2DM. This study was conducted to determine the impact of the interaction between thyroid disorders, including hypothyroidism and/or thyroid autoimmunity, and GDM on the incidence of T2DM among participants of a population-based cohort study, the Tehran Thyroid Study (TTS), over a median follow up of 12.5 years.

## Methods

### Study participants

We selected participants from the population of the Tehran Thyroid study (TTS), which is a population-based prospective study that is within the framework of the Tehran lipid and glucose study (TLGS) [12]. Tehran lipid and glucose study is a population-based cohort study conducted on 15,000 individuals who live in the district 13 of Tehran (capital of Iran), aimed to evaluate the prevalence and incidence of risk factors for non-communicable diseases [13].

In the TTS, 5783 individuals (3407 women and 2376 men) aged ≥20 years were randomly selected between March 1997–December 2004 [14]. Each participant was invited to the TLGS unite every 3 years; A general physical examination and information regarding the reproductive history and pregnancy outcomes were recorded. Details of the study have been published elsewhere [14].

**Fig. 1:**
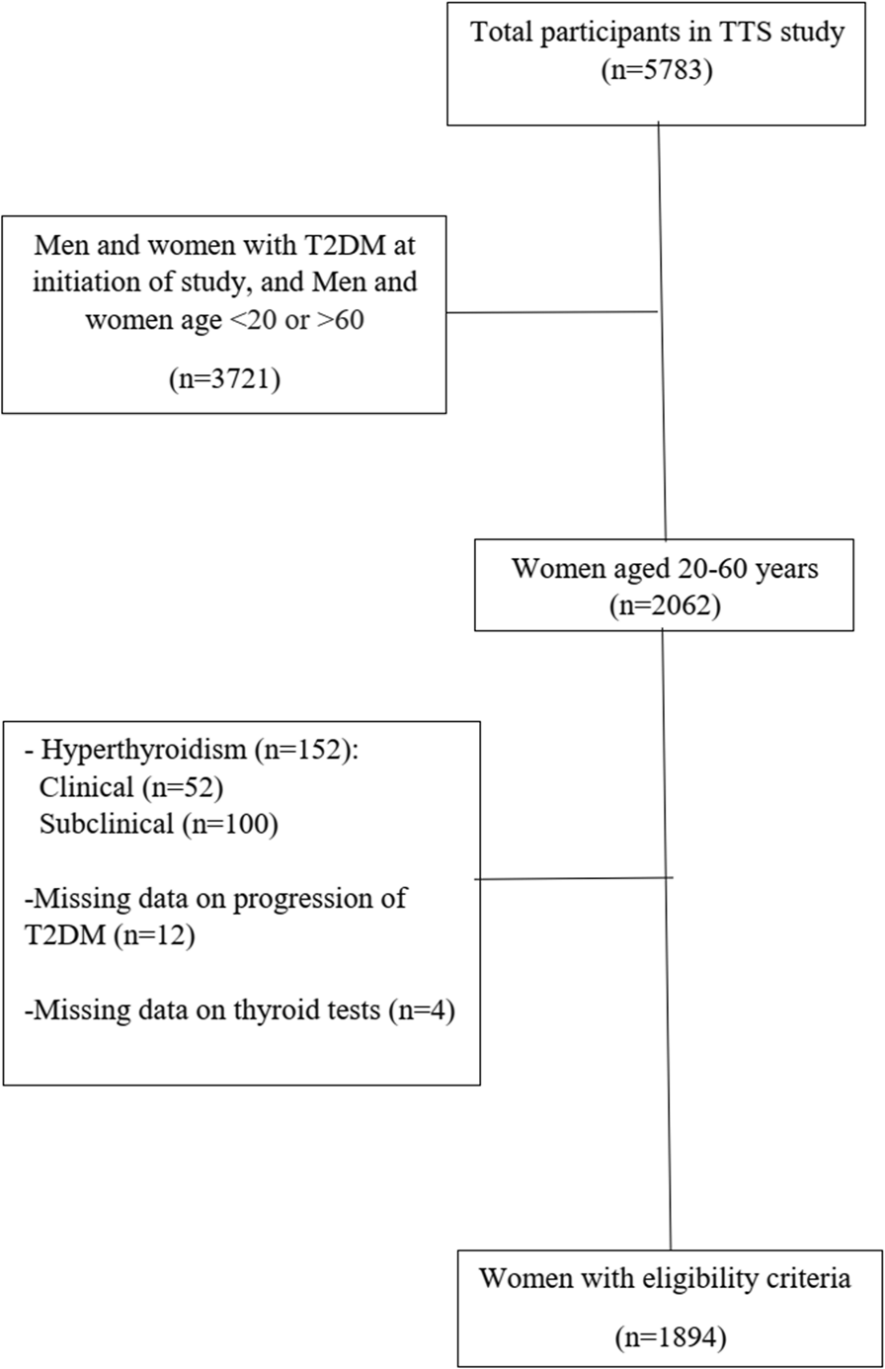
Overview of the study population

In the current study, those women with at least one follow up visit and term delivery without known T2DM at the baseline were included (*n* = 2062). Participants with a diagnosis of hyperthyroidism (*n* = 152) and missing data (*n* = 16) were also excluded. Finally, data of 1894 eligible participants were used in the current analyses; the study flowchart is presented in Fig. 1.

The study proposal was approved by the medical ethics committee of the Research Institute for Endocrine Sciences (RIES) and written informed consent was obtained from all participants.

### Clinical measurements

All participants invited to the TTS were referred to trained physicians after giving written informed consent. Details of examinations and procedures have been previously published [14]. In brief, weight and height were measured, and the body mass index (BMI) of participants was calculated. Blood pressure was measured twice with a standard mercury sphygmomanometer.

### Laboratory measurements

Blood samples were taken after a 12 to 14 h overnight fast from all study participants. Participants who were not taking glucose-lowering drugs were examined with 75 g oral glucose tolerance test (OGTT). Furthermore, fasting and 2-h plasma glucose (2hPG) were evaluated. Serum total cholesterol (TC) and triglycerides (TGs) were measured using the enzymatic colorimetric method [13]. Baseline and follow-up free thyroxine (FT4) and thyroid-stimulating hormone (TSH) were measured by the electrochemiluminescence immunoassay method. Thyroid peroxidase antibody (TPOAb) was determined by immunoenzymometric assay (IEMA) [12]. All measurements were simultaneously performed at the research laboratory of the Research Institute for Endocrine Sciences (RIES).

### Definition of variables and outcomes

All females were asked through an interview about their history of GDM based on a self-reporting questionnaire and medical records if needed [15]. We used the World Health Organization (WHO) criteria for universal screening of GDM according to the Iranian national screening program [16].

For the definition of T2DM, we apply The American Diabetes Association [17] criteria: using anti-diabetic drugs or having FBS ≥ 7 mmol/l (measured twice) or 2 h plasma glucose (OGTT) ≥11.1 mmol/l. Having a history of T2DM in the 1st-degree family members was considered as a family history of diabetes. Hypothyroidism were defined as serum TSH level > 5.06 mU/L or FT4 < 0.91 ng/dl [18]. TPOAb positivity was defined as having values > 35.04 IU/mL in females [19]. In the present study, we defined thyroid disorders as the presence of hypothyroidism or TPOAb positivity or using Levothyroxine. We excluded all those with hyperthyroidism as well as those using anti-thyroid drugs.

### Statistical analyses

Continuous variables were checked for normality using the one-sample Kolmogorov–Smirnov test and reported as mean (standard deviation) if they had a normal distribution or median with inter-quartile range (IQR25–75) for variables with skewed distribution. Categorical variables are presented as numbers and percentages. Characteristics of women were compared between groups, applying the ANOVA or *x*^2^ test for continuous and categorical variables, respectively. The Kruskal-Wallis test was applied to compare variables with skewed distribution. As data was interval censored and time to T2DM was not known, pooled logistic regression was used to assess the impact of GDM, thyroid disorders, free T4, TSH, thyroid autoimmunity and the interaction between GDM and thyroid disorders on developing T2DM as well as to calculate odds ratios (OR). This model treats every interval as a mini follow-up study, pools the observations of all intervals together into one pooled sample, and does a logistic regression on the pooled dataset [20].

All analyses were adjusted for age (model 1); age, BMI, educational status, smoking, family history of DM (model 2) and intermediate covariates, including serum TG and TC concentration, systolic blood pressure (SBP), diastolic blood pressure (DBP), and FBS (model 3). Statistical analysis was performed using the software package STATA (version 14; STATA Inc., College Station, TX, USA); significance level was set at *P* < 0.05, and confidence interval (CI) as 95%.

## Results

Of 1894 eligible women, 346 (18.3%) had GDM, and 493 (26%) had thyroid disorders, including hypothyroidism and/or thyroid autoimmunity; of those with thyroid disorders, 77 (15.6%) had a history of GDM, as well. The median and IQR for follow-up years were 12.5 (IQR: 11.8–13.4).

Subjects were divided into four groups based on having a history of GDM and thyroid disorders as follows: 1) not having a history of GDM and thyroid disorders; 2) having a history of GDM and thyroid disorders; 3) having a history of GDM without thyroid disorders and 4) having thyroid disorders without a history of GDM.

Baseline characteristics of study participants based on their history of GDM and thyroid disorders are presented in Table 1. Individuals without a history of GDM and thyroid disorders had lower BMI compared to other groups. We found significant differences in FT4, TSH, and TPOAb levels between groups.

**Table 1:** Characteristics of the study participants at recruitment

Group	Without GDM & without thyroid disorders(***N*** = 1132)	With GDM & with thyroid disorders(***N*** = 77)	With GDM & without thyroid disorders(***N*** = 269)	Without GDM & with thyroid disorder(***N*** = 416)	P-value
**Variables**					
Age (Years)^a^	39.06 ± 10.06	36.06 ± 8.20	38.33 ± 7.71	40.31 ± 10.22	0.07
Weight (Kg)^a^	67.22 ± 10.76	73.52 ± 12.28	71.44 ± 11.27	69.71 ± 11.75	< 0.001
Height(m)^a^	1.56 ± 0.0538	1.57 ± 0.05	1.57 ± 0.05	1.57 ± 0.05	0.05
BMI (kg/ *m*^2^)^a^	27.34 ± 4.34	29.74 ± 5.18	28.62 ± 4.33	28.20 ± 4.57	< 0.001
FBS (mg/dL)^a^	88.46 ± 8.70	87.74 ± 10.11	89.73 ± 10.09	87.67 ± 8.69	0.03
BS-2hpp(mg/dL)^a^	106.62 ± 25.82	107.76 ± 24.80	109.07 ± 22.89	104.12 ± 24.60	0.08
Smoking status	39 (3.4%)	2 (2.6%)	14 (5.2%)	20 (4.8%)	0.37
Family history of diabetes	316 (27.9%)	21 (27.3%)	80 (29.7%)	101 (24.3%)	0.44
Educational status					0.02
< 6 years	384 (33.9%)	32 (41.5%)	69 (26.6%)	122 (29.3%)	
6–12 years	632 (55.8%)	41 (53.2%)	178 (66.2%)	256 (61.5%)	
> 12 years	116 (10.2%)	4 (5.2%)	22 (8.2%)	38 (9.1%)	
Triglycerides (mg/dL) ^b^	126 (87–181)	121 (85–176)	128 (92–201)	131 (90–189)	0.24
Total Cholesterol (mg/dL)^a^	205.14 ± 46.14	200.18 ± 37.10	205.21 ± 38.00	209.60 ± 47.56	0.22
Systolic-BP (mmHg)^a^	114.06 ± 16.87	111.66 ± 13.49	113.04 ± 13.93	115.14 ± 16.35	0.21
Diastolic-BP (mmHg)^a^	76.08 ± 10.42	74.93 ± 8.30	76.53 ± 9.75	75.93 ± 10.18	0.66
Free-T4(ng/dL)^b^	1.16 (1.06–1.26)	1.02 (0.86–1.20)	1.13 (1.06–1.24)	1.05 (0.89–1.18)	< 0.001
TSH (mIU/L)^b^	1.72 (1.09–2.60)	3.59 (1.87–6.33)	1.61 (1.08–2.31)	3.90 (1.98–6.37)	< 0.001
TPOAb level (IU/mL)^b^	5.05 (3.17–8.39)	44.49 (4.94–264.77)	4.33 (3.15–9.52)	71.58 (12.49–245.28)	< 0.001
Hypothyroidism n(%)^c^	0	10 (13%)	0	54 (13%)	< 0.001
Subclinical Hypothyroidism n(%)^c^	0	17 (22.1%)	0	113 (27.2%)	< 0.001
TPOAb positivity n(%)^c^	0	47 (61%)	0	274 (65.8%)	< 0.001
Hypothyroxinemia n(%)^c^	0	15 (19.5%)	0	59 (14.2%)	< 0.001

^a^ Variables with normal distribution are reported as Mean ± SD and compared using ANOVA test

^b^ Variables with non-normal distribution are reported as median (Q1, Q3) compared using Kruskal Wallis Test

^c^ Categorical variables are presented as number (%) and compared using the chi-square test

*FBS* Fasting blood sugar, *Bs-2hpp* Blood sugar 2 h postprandial, *Systolic BP* Systolic blood pressure, *Diastolic BP* Diastolic blood pressure, *Free-T4* Free thyroxine, *TSH* Thyroid-stimulating hormone, *TPOAb* Thyroid peroxidase antibody

The results of pooled logistic regression analyses on the association of thyroid disorders, GDM, and interaction terms of thyroid disorders and GDM for developing T2DM are presented in Table 2. Compared to women without a history of GDM, OR for the T2DM incident was significantly higher among individuals with GDM in an unadjusted model (OR: 1.54; 95%CI: 1.06, 2.25); the association observed remained statistically significant even after adjusting for age (OR: 1.67; 95%CI: (1.14, 2.45). However, this association lost significance after further adjustment for BMI, educational status, smoking, and family history of DM, TG, TC, HDL-C, SBP, DBP, and FBS. Our study found no significant association between either thyroid disorders (OR: 1.14, 95%CI: 0.82, 1.58) or the interaction between GDM and thyroid disorders (OR: 1.27, 95%CI: 0.66, 2.43) on development of T2DM.

**Table 2:** Associations of GDM, thyroid disorders, and interaction between GDM and thyroid disorders on development of T2DM using a pooled logistic analysis

Variables	Unadjusted model		Model 1		Model 2		Model 3	
	OR (95% CI)	P-value	OR (95% CI)	P-value	OR (95% CI)	P-value	OR (95% CI)	P-value
**Thyroid disorder**	1.14 (0.82, 1.58)	0.44	1.13 (0.81, 1.57)	0.46	1.08 (0.77, 1.51)	0.64	1.24 (0.87, 1.75)	0.22
**GDM**	1.54 (1.06, 2.25)	0.02	1.67 (1.14, 2.45)	0.01	1.40 (0.94, 2.07)	0.09	1.42 (0.95, 2.12)	0.08
**GDM × Thyroid disorder**	1.27 (0.66, 2.43)	0.47	1.32 (0.69, 2.54)	0.40	1.16 (0.58, 2.31)	0.67	1.41 (0.71, 2.80)	0.33

Model 1: adjusted for age

Model 2: adjusted for age, BMI, educational status, smoking and family history of DM

Model 3: adjusted for serum triglycerides, serum total-cholesterol, serum HDL-cholesterol, systolic blood pressure, diastolic blood pressure, and fasting plasma glucose

Thyroid disorders: Clinical, subclinical hypothyroidism, and thyroid autoimmunity, GDM: gestational diabetes, T2DM: type 2 diabetes mellitus,

*OR* odds ratio, *CI* confidence interval

Results of pooled logistic regression analyses on the impact of GDM, serum FT4 levels, and the interaction between GDM and serum FT4 concentration on the development of T2DM are shown in Table 3. There was no significant association of the interaction between GDM and FT4 with the progression of T2DM; neither was any relation found between serumFT4, TSH, and TPOAb levels and the incidence of T2DM in pooled logistic regression analyses before and after adjustment for potential confounders (Table 3 and supplementary Tables 1, and 2).

**Table 3:** Associations of GDM, serum FT4 levels, and interaction between GDM and serum FT4 on the development of T2DM using pooled logistic regression analyses

Variables	Unadjusted model		Model 1		Model 2		Model 3	
	OR (95% CI)	P-value	OR (95% CI)	P-value	OR (95% CI)	P-value	OR (95% CI)	P-value
**FT4(ng/dL)**	1.02 (0.94, 1.10)	0.70	1.02 (0.94, 1.11)	0.63	1.01 (0.93, 1.10)	0.76	1.0.6 (0.97, 1.15)	0.23
**GDM**	1.05 (0.15, 7.10)	0.96	0.95 (0.13, 6.67)	0.96	0.42 (0.06, 3.07)	0.39	0.58 (0.08, 4.35)	0.60
**GDM × FT4**	0.95 (0.81, 1.13)	0.60	0.94 (0.79, 1.11)	0.48	0.89 (0.75, 1.06)	0.19	0.91 (0.77, 1.09)	0.31

Model 1: adjusted for age

Model 2: adjusted for age, BMI, educational status, smoking and family history of DM

Model 3: adjusted for serum triglycerides, total and HDL-cholesterol levels, systolic blood pressure, diastolic blood pressure, and FBS

*GDM* gestational diabetes, *T2DM* type 2 diabetes mellitus, *FT4* free thyroxin

The total cumulative incidence rate of T2DM diagnosed at the median follow-up time of 12.5 years was overall 12/1000 person-years (95%CI: 10/1000–13/1000), with an incidence rate of 16/1000 (95%CI: 13/1000–20/1000) in women with GDM; it was 11/100,000 (95%CI: 9/100,000–12/1000) among those without GDM. The cumulative incidence rate of T2DM for women with and without thyroid disorders was 13/1000 (95%CI: 10/1000–16/1000) and 11/1000 (95%CI: 10/1000–13/1000), respectively.

We checked the correlation between age and hypothyroidism via a logistic regression model with hypothyroidism as a binary outcome variable. We got an OR of 1.02 (95% CI: 0.99–1.04) for the age variable, which was not statistically significant (*p* = 0.05). In the same way, we did not get any significant association between age and GDM (OR = 0.99, 95%CI = (0.97–1.01), *p*-value = 0.300). We also considered the interaction term between age and GDM; however, the result was not significant (OR = 0.96, 95% CI: 0.89–1.04, *p*-value = 0.4).

## Discussion

The present study indicated no synergistic impact of GDM with thyroid disorders on the incidence of T2DM. Despite the higher risk of incident T2DM among women with a history of GDM, women with thyroid dysfunction had similar risk for development of T2DM, compared to their counterparts with normal thyroid function. Therefore, women with a history of GDM should be followed up regarding a high risk of T2DM beyond their experience of thyroid disorders.

To the best of our knowledge, no previous studies have investigated the synergistic impact of GDM with thyroid disorders on the development of T2DM. However, several have investigated the associations of GDM and thyroid disorders per se with the risk of incident T2DM [20–23]. The findings of our study showed a higher risk of developing T2DM in patients with a history of GDM, compared to those without a history of GDM, an observation consistent with the findings of previous studies [20–23]. In a very recent meta-analysis, the subsequent risk of T2DM in women with GDM compared to those without GDM was 9.51 (95% CI: 7.14 to 12.67) Another meta-analysis of cohort studies conducted by Bellamy et al. revealed that the risk of incident T2DM in women with a history of GDM was 7.43 (95%CI:4.79–11.51) times higher than those without any history [23]. The adverse association of GDM with developing T2DM may be attributable to epigenetic changes induced by maternal hyperglycemia in target tissues, such as skeletal muscle and subcutaneous adipose tissue [24]. Moreover, increase in circulating levels of leptin, inflammatory biomarkers e.g., TNF-α and C-reactive protein (CRP) and the fat content in liver and muscle, as well as the decreased adiponectin concentration reported in women with prior GDM, may partly explain woman’s predisposition to T2DM following GDM [25].

It should be noted that we observed a significant association between GDM and higher subsequent T2DM risk after adjusting for age, but this association became attenuated and non-significant after further adjustment for BMI, educational status, smoking and family history of DM, TG, TC, HDL-C, SBP, DBP, and FBS. The major reduction in the effect size was observed after controlling for BMI, educational status, smoking, and family history of DM. It can be explained by the positive association of BMI with the development of T2DM among women with a history of GDM. Accumulation of fat in body and excess weight are well-defined risk factors for T2DM in a population [26, 27]. On the other side, women experiencing GDM are more prone to weight gain at the onset of elevated FBS and developed GDM as well as a higher probability for the development of T2DM in later life [28]. The presence of GDM is a hallmark to make a recommendation to monitor their weight after delivery [28]. Therefore, the association between GDM and incidence of T2DM could partly be attributed to the BMI. The same cofounding effect could exist for other adjusted covariates, especially for smoking and family history of DM*.*

There is no consensus about the adverse influence of thyroid dysfunction on the further development of T2DM. The present study showed no statistically significant increase in the development of T2DM following thyroid disorders. In agreement with the results of our research, Sadatamini et al. showed that the incidence of thyroid dysfunction in T2DM patients was not higher than non-diabetic participants during 12 years of follow up [29]. However, a longitudinal cohort study conducted among 25,575 adults aged > 18 years by Chen et al. in Taiwan with follow up of > 10 years reported that the incidence of T2DM was higher among individuals with either hypo- or hyperthyroidism, with most incident cases of T2DM occurring the first five years of thyroid disorders [30]. In another prospective cohort of 8452 participants with a 7.9 year follow up, hypothyroidism was identified as a risk factor for increased risk of incident T2DM, more so in pre-diabetic patients [6], findings contrary to those of our investigation; their findings however also indicated that the risk for developing T2DM drops from 35 to 15% when FT4 reached to normal levels [6]. Thyroid hormones have a regulatory function on carbohydrates metabolism [31]. Thus, impairment in thyroid regulation had an undesirable impact on carbohydrate metabolism and glucose homeostasis s through their direct effect on the control of insulin secretion and the preservation of beta-cell proliferation and viability [32, 33]. Increased higher FT3 levels are associated with impaired glucose tolerance through elevated liver gluconeogenesis and the development of insulin resistance [34]. However, evidence indicated that the incidence of T2DM in patients with hyperthyroidism was only 2 to 3.3% [30]. Interestingly, thyroxine intervention in patients with diagnosed hypothyroidism decreased HbA1c, independent of changes in plasma glucose [35]. Besides, the value of HbA1c patients with hypothyroid was higher in comparison to those in the control group [36]. This observation was also beyond the level of FPG. It seems that there is a very high false-positive rate for HbA1c among patients with hypothyroidism.

No significant associations of serum FT4, TSH, and TPOAb levels with incidence of T2DM were observed in our data, using either simple or pooled logistic regression analyses. In contrast of our findings, Yeqing et al. in a cross-sectional study (*n* = 15,296), performed in China, demonstrated that decreased FT3, FT3/FT4 ratios, and increased FT4 levels are independently related to a higher prevalence of T2DM in both males and females, and TSH is inversely associated with T2DM in males only [37]; however the results of the Mohammed et al. study of 2797 type 2 diabetic patients, revealed no significant differences in serum thyroid levels between T2DM patients and their healthy counterparts (*P* < 0.05); their results also indicated that the frequency of thyroid autoimmunity was not significantly higher in type 2 diabetic patients than in the non-diabetic control group [38].

According to the results of the current study, the incidence of T2DM increased significantly with time and age, a finding in agreement with the results of previous studies [39–41]. Evidence suggests that senescent cells, implicated in the generation of insulin resistance, accumulate in various tissues with aging [42].

Regarding study strengths, this is the first study with a longitudinal design, long term follow-up, and large sample size investigating the possible interaction between the history of GDM and thyroid disorders in the incidence of T2DM. Also, using pooled logistic approaches helped us to further adjust our results for assumed confounders that were precisely measured in this study including age, familial history of diabetes, smoking, anthropometric indices, and lipid profiles. However, the present analyses do have some limitations, which should be addressed. First, GDM was determined based on a self-reporting questionnaire and medical records, although the universal screening strategy of GDM in Iran and subsequent monitoring and treatment of GDM may restrict this bias. Our study is limited by lack of data on some parameters such as insulin use that may affect the progression to T2DM. Furthermore, Of 1894 participants of the present study, 146 (7.7%) of whom had hypothyroidism. Unfortunately, there was not enough power to check the interaction term between hypothyroidism and age, which was the limitation of this study. Last but not least, serum FT3 levels, as biologically active hormones involved in glucose metabolism were not assessed in the current study.

## Conclusion

In conclusion, no synergistic association was found between GDM and thyroid disorders on the incidence of T2DM. However, women with a history of GDM were at higher risk of developing T2DM later in their life. As women age, the risk of T2DM incidence is also increasing. Given the significant prevalence of T2DM, targeted healthcare systems, and lifestyle modification are recommended.

## Supplementary information

**Additional file 1: Supplementary Table 1.** Associations of GDM, serum TSH levels, and interaction between GDM and serum TSH on the development of T2DM using pooled logistic regression analyses. **Supplementary Table 2.** Associations of GDM, serum TPOAb levels and interaction between GDM and serum TPOAb on development of T2DM using pooled logistic regression analyses.

## Data Availability

The datasets analyzed during the current study are available from the corresponding author on reasonable request.

## Abbreviations

GDM: Gestational diabetes
T2DM: Type 2 diabetes
TTS: Tehran Thyroid Study
TLGS: Tehran lipid and glucose study
FT4: Free thyroxin
TSH: thyroid stimulating hormone
TPOAb: Thyroid peroxidase antibody
RIES: Research Institute for Endocrine Sciences
OGTT: Oral glucose tolerance test
2hPG: 2-h plasma glucose
TC: Serum total cholesterol
TGs: serum triglycerides
SBP: Systolic blood pressure
DBP: diastolic blood pressure
