# Supplementary material for: Assessment of the simultaneous effect of hypothyroidism and thyroid autoimmunity with gestational diabetes on the incidence of type 2 diabetes
Source: BMC Endocr Disord. 2020 Oct 1;20:150. doi: 10.1186/s12902-020-00627-z (PMC7528385; doi:10.1186/s12902-020-00627-z)
Supplement: Supplementary file 1 — Additional file 1: Supplementary Table 1. Associations of GDM, serum TSH levels, and interaction between GDM and serum TSH on the development of T2DM using pooled logistic regression analyses. Supplementary Table 2. Associations of GDM, serum TPOAb levels and interaction between GDM and serum TPOAb on development of T2DM using pooled logistic regression analyses. [file 12902_2020_627_MOESM1_ESM.docx]

| **Supplementary Table 1. Associations of GDM, serum TSH levels, and interaction between GDM and serum TSH on the development of T2DM using pooled logistic regression analyses.** | | | | | | | | |
| --- | --- | --- | --- | --- | --- | --- | --- | --- |
| **Variable** | **Unadjusted model** | | **Model 1** | | **Model 2** | | **Model 3** | |
|  | **OR (95% CI)** | **P-value** | **OR (95% CI)** | **P-value** | **OR (95% CI)** | **P-value** | **OR (95% CI)** | **P-value** |
| **TSH (mIU/L)** | 0.99(0.98, 1.01) | 0.95 | 1.00(0.98, 1.01) | 0.98 | 1.00(0.98, 1.02) | 0.99 | 1.00(0.99, 1.02) | 0.49 |
| **GDM** | 2.04(1.33, 3.13) | 0.001 | 2.21(1.46, 3.36) | 0.001> | 1.82(1.17, 2.82) | 0.008 | 1.92(1.24, 2.98) | 0.003 |
| **GDM *TSH** | 0.94(0.83, 1.06) | 0.31 | 0.94(0.84, 1.06) | 0.32 | 0.94(0.83, 1.06) | 0.29 | 0.94(0.83, 1.06) | 0.31 |
| Model 1: adjusted for age,  Model 2: adjusted for age, BMI, educational status, smoking and family history of DM,  Model 3: adjusted for serum triglycerides, total and HDL-cholesterol levels, systolic blood pressure, diastolic blood pressure, and FBS. | | | | | | | | |
| GDM: Gestational diabetes, T2DM: Type 2 diabetes mellitus, TSH: Thyroid-stimulating hormone | | | | | | | | |

| **Supplementary Table 2. Associations of GDM, serum TPOAb levels and interaction between GDM and serum TPOAb on development of T2DM using pooled logistic regression analyses.** | | | | | | | | |
| --- | --- | --- | --- | --- | --- | --- | --- | --- |
| **Variables** | **Unadjusted model** | | **Model 1** | | **Model 2** | | **Model 3** | |
|  | **OR (95% CI)** | **P-value** | **OR (95% CI)** | **P-value** | **OR (95% CI)** | **P-value** | **OR (95% CI)** | **P-value** |
| **TPOAb (IU/mL)** | 1.00(0.99, 1.00) | 0.82 | 1.00(2.67, 6.60) | 0.78 | 0.99(0.99, 1.00) | 0.99 | 1.00(0.99, 1.00) | 0.29 |
| **GDM** | 1.65(1.20, 2.27) | 0.00 | 1.82(1.32, 2.51) | 0.001> | 1.47(1.04, 2.06) | 0.02 | 1.56(1.11, 2.20) | 0.01 |
| **GDM *TPOAb** | 1.00(0.99, 1.00) | 0.31 | 1.00(0.99, 1.00) | 0.37 | 1.00(0.99, 1.00) | 0.43 | 1.00(0.99, 1.00) | 0.39 |
| Model 1: adjusted for age,  Model 2: adjusted for age, BMI, educational status, smoking and family history of DM,  Model 3: adjusted for serum triglycerides, total and HDL-cholesterol levels, systolic blood pressure, diastolic blood pressure, and FBS. | | | | | | | | |
| GDM: Gestational diabetes, T2DM: Type 2 diabetes mellitus, TPOAb: Thyroid peroxidase antibody | | | | | | | | |
